# A dynamic histone-based chromatin regulatory toolkit underpins genome and developmental evolution in an invertebrate clade

**DOI:** 10.1186/s13059-025-03626-2

**Published:** 2025-06-10

**Authors:** Francisco M. Martín-Zamora, Joby Cole, Rory D. Donnellan, Kero Guynes, Allan M. Carrillo-Baltodano, Mark J. Dickman, Paul J. Hurd, José M. Martín-Durán

**Affiliations:** 1 School of Biological and Behavioural Sciences, Queen Mary University of London, Mile End Road, London, E1 4 NS UK; 2 Altos Labs, Cambridge Institute of Science, Granta Park, Cambridge, CB21 6GP UK; 3 Department of Infection and Tropical Medicine, Sheffield Teaching Hospitals NHS Foundation Trust, Royal Hallamshire Hospital, Glossop Road, Sheffield, S10 2JF UK; 4 Department of Infection, Immunity and Cardiovascular Disease, School of Medicine and Population Health, University of Sheffield, 387 Glossop Road, Sheffield, S10 2HQ UK; 5 Institute of Molecular Biotechnology of the Austrian Academy of Sciences (IMBA), Vienna BioCenter (VBC), Vienna, 1030 Austria; 6 Department of Chemical & Biological Engineering, School of Chemical, Materials and Biological Engineering, University of Sheffield, Mappin Street, Sheffield, S1 3JD UK

**Keywords:** Histone, Histone posttranslational modifications, Histone-modifying enzyme, Animal development, Annelida, Spiral cleavage, Evolution, H2A.X

## Abstract

**Background:**

The dynamic addition and removal of posttranslational modifications on eukaryotic histones define regulatory regions that play a central role in genome and chromatin biology. However, our understanding of these regulatory mechanisms in animals is primarily based on a few model systems, preventing a general understanding of how histone-based regulation directs and promotes phenotypic variation during animal embryogenesis.

**Results:**

Here, we apply a comprehensive multi-omics approach to dissect the histone-based regulatory complement in Annelida, one of the largest invertebrate clades. Annelids exhibit a conserved histone repertoire organized in clusters of dynamically regulated, hyperaccessible chromatin. However, unlike other animals with reduced genomes, the worm *Dimorphilus gyrociliatus* shows a dramatically streamlined histone repertoire, revealing that genome compaction has lineage-specific effects on histone-based regulation. Notably, the annelid *Owenia fusiformis* has two H2A.X variants that co-occur in other animals, sometimes associate with fast cell divisions, and represent a unique case of widespread parallel evolution of a histone variant in Eukarya. Histone-modifying enzyme complements are largely conserved among annelids. Yet, temporal differences in the expression of a reduced set of histone modifiers correlate with distinct ontogenetic traits and variation in the adult landscapes of histone posttranslational modifications, as revealed by quantitative mass spectrometry in *O. fusiformis* and *Capitella teleta*.

**Conclusions:**

Our analysis of histone-based epigenetics within a non-model phylum informs the evolution of histone-based regulation, presenting a framework to explore how this fundamental genome regulatory layer generally contributes to developmental and morphological diversification in annelids and animals.

**Supplementary Information:**

The online version contains supplementary material available at 10.1186/s13059-025-03626-2.

## Background

**Fig. 1:**
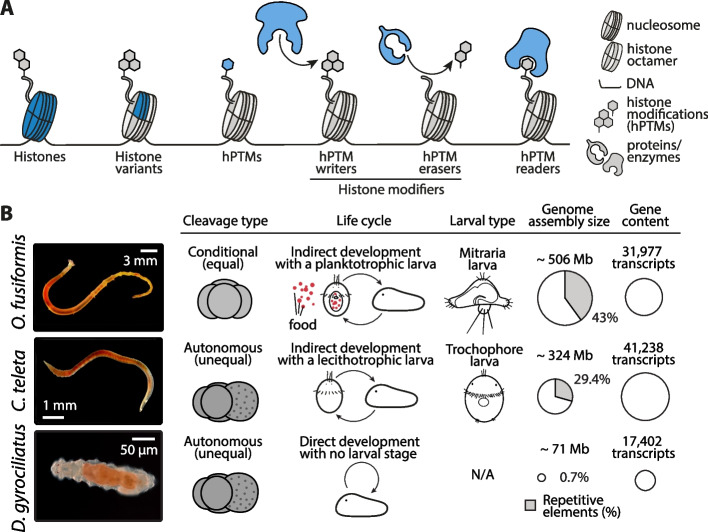
Histone-based regulation of three annelid species.** A** Schematic drawing depicting the potential sources of evolutionary variation in the histone-based regulation complement. Drawings are not to scale. **B** *O. fusiformis*, *C. teleta*, and *D. gyrociliatus* possess a very conserved early embryogenesis program yet display key differences. From left to right, cleavage type, life cycle, larval type, genome assembly size and repetitive elements percentage, and gene content are displayed for each species. While *D. gyrociliatus* and *C. teleta* are unequal cleavers and commit cell fates during the first cell divisions, *O. fusiformis* is a conditional cleaver that does not do so until later cleavage stages. *O. fusiformis* possesses the mitraria larva, a feeding, i.e., planktotrophic, larva; while *C. teleta* is an example of a species with a classic non-feeding or lecithotrophic trochophore larva, and *D. gyrociliatus* lacks a larval stage. *D. gyrociliatus* has a very compact genome, almost devoid of repetitive elements, that has reduced its size and gene content conservatively. Images are for adult specimens of each species. Bubble plots are proportional to genome size and gene content. hPTMs: histone posttranslational modifications

Genome packaging is a critical phenomenon in cell biology. Different mechanisms, like DNA supercoiling, aid in this process [1], yet the packaging in particular viruses [2, 3], bacteria [4], archaea [5], and especially in eukaryotes [6–8], results from the interaction of genomic DNA with histones. Eukaryotic histones are highly basic, DNA-associated proteins that protect and package nuclear naked DNA into higher-order, densely compacted structures up to the chromosome level. By doing so, they play a central role as regulators of genome architecture and almost all DNA-based biological processes, especially the regulation of gene expression [6, 9, 10]. Histone-based regulation is one of the most versatile and intricate mechanisms modulating genome function [9, 11, 12] (Fig. 1A). These mechanisms include histone dynamics and differential usage of histone variants [13–15]. Yet, arguably, the most functionally crucial regulatory mechanism involves the deposition and removal of small covalent posttranslational modifications, such as methyl and acetyl moieties, to key residues primarily located in the N-terminal end, or tail, of histones [16, 17]. Specific histone posttranslational modifications (hPTMs) in the core histone regions cause structural perturbations in the nucleosome structure, thereby changing the level of chromatin condensation or accessibility. Others act as binding marks for modules of reader proteins and complexes that recruit chromatin-modifying machinery, leading to different biological readouts [9, 12, 18–22]. In this way, hPTMs define regulatory regions in the genome and their levels of regulatory activity, constituting critical regulators of various processes like proliferation, differentiation, and most importantly, embryogenesis and development [12, 23–26]. Even though the central histone-based regulation mechanisms are largely evolutionarily conserved, their vast regulatory activity makes them a promising source of evolutionary variation.

Little is known about the repertoire and role of hPTMs outside traditional animal models for which high-quality reference genomes and methods to interrogate histone regulation have been readily available. Functional genomics work has been limited in Spiralia, one of the three largest clades of bilaterally symmetrical animals comprising morphologically diverse lineages and nearly half of the animal phyla [27, 28]. Research on histones peaked during the second half of the twentieth century. Those early works were primarily limited to biochemical studies on histone sequence, structure, and composition differences across a handful of major groups, namely molluscs [29–32], annelids [33–35], and nemerteans [36]. Likewise, hPTM-targeting functional genomics studies with chromatin immunoprecipitation combined with high-throughput sequencing (ChIP-seq) [37, 38] and more modern techniques [39–43] have so far been restricted to Platyhelminthes. Within this phylum, efforts have focused on understanding stem cell maintenance and regeneration in the planarian *Schmidtea mediterranea* [44–52], and the life cycle of the parasitic helminth *Schistosoma mansoni* to develop potential treatments to schistosomiasis, the disease it causes [53–61]. Therefore, our knowledge of histone-based regulation in non-model systems such as spiralians is quite limited, thus hampering a comprehensive understanding of how changes in genome regulation promote variation in gene expression programs and, ultimately, phenotypic change during animal evolution.

Variations in the timing or rate of development, commonly called heterochronies, are a key source of evolutionary variation [62–65]. In annelids, one of the largest spiralian clades, and likely in bilaterians generally [66–68], developmental heterochronies correlate with the diversification of embryonic outcomes and life cycles. The annelids *Owenia fusiformis* Delle Chiaje, 1844; *Capitella teleta* Blake, Grassle & Eckelbarger, 2009; and *Dimorphilus gyrociliatus* (O. Schmidt, 1857) share a common early developmental program—spiral cleavage—but have distinct strategies for cell fate specification, life cycles, and larval types (Fig. 1B) [69–71]. In these annelids, genes involved in key cellular processes (e.g., autophagy) and enzymatic pathways (e.g., chitin synthesis pathway) are either pre- or post-displaced between larval types, i.e., they have an earlier or later expression onset, respectively [66]. Moreover, the trunk patterning transcriptional program, which includes the expression of the *Hox* genes, is progressively brought forward in development—i.e., pre-displaced—as larval traits are lost and indirect development transitions into direct development [66]. Importantly, these changes in gene expression mirror chromatin accessibility dynamics, as observed in the open chromatin regions of the *Hox* cluster and the transcription factor binding dynamics of HOX DNA-binding motifs [66]. Therefore, given their fundamental role in regulating gene expression, differences in the dynamics of histone-based regulation—particularly of hPTMs—may be upstream and account for these heterochronies, providing a mechanistic explanation of how changes in genome regulation trigger adaptive variation in developmental programs.

Here, we begin to tackle the relationship between histone-based regulation and developmental transcriptional programs by mining and characterizing the histone complement and histone modification machinery in the annelids *O. fusiformis*, *C. teleta*, and *D. gyrociliatus* through a large-scale multi-omics profiling at the genomic, transcriptomic, epigenomic, and proteomic levels. We describe a minimal histone complement in the miniature genome of *D. gyrociliatus* and identify divergence in a key histone variant throughout Eukarya that may account for the evolution of early embryonic phenotypes in animals. Despite their differences in histone numbers, the repertoires of histone modifiers are mainly complete and conserved between these annelids. However, some families exhibit expansions and domain fusions that suggest neofunctionalization events. Concomitant with the heterochronies observed in other gene regulatory networks and pathways, some histone modifiers exhibit shifts in their developmental expression times between these annelids, correlating with life cycle and larval type differences, as well as with the levels of histone posttranslational modifications (hPTMs) in the adults. Altogether, our study is the most comprehensive comparative characterization of histone-based regulation in a spiralian clade, paving the way for the genome-wide profiling of hPTMs in annelids and the functional assessment of the interplay between histone modifications and developmental programs in the phenotypic diversification of this animal group.

## Results

### Annelids exhibit a conserved histone repertoire

**Fig. 2:**
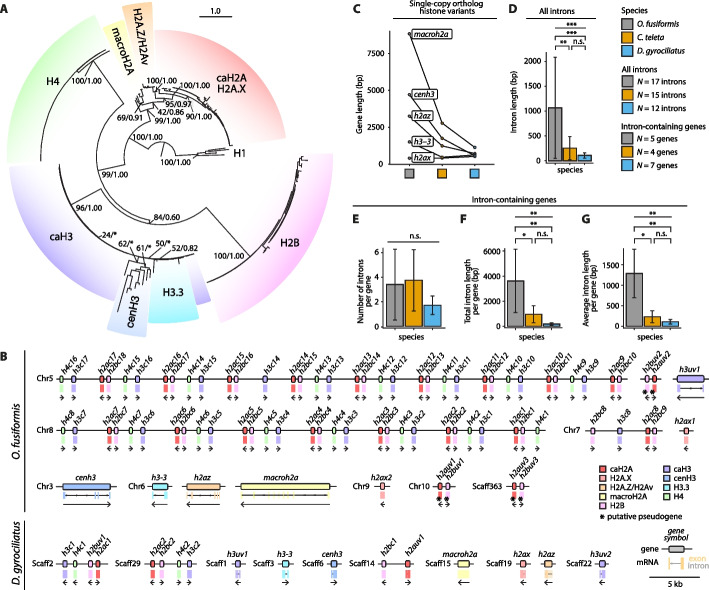
Annelids exhibit a conserved histone repertoire. **A** Summary phylogeny of the gene orthology analysis of histone genes in *O. fusiformis*, *C. teleta*, and *D. gyrociliatus*. Depicted tree topology is based on the maximum likelihood reconstruction. Branch support values represent bootstrap values (0–100 values) and posterior probabilities (0–1 values) at key nodes. Colored boxes highlight the extent of each histone gene or family. Clades supported by maximum likelihood only are flagged with an asterisk (*). The scale bar depicts the number of amino acid changes per site along the branches. caH2A: canonical H2A; caH3: canonical H3; cenH3: centromeric H3. **B** Schematic representation to scale of the genomic loci of the histone genes in *O. fusiformis* (in the chromosome-level assembly) and in *D. gyrociliatus*. Boxes delimitate gene bodies, with the intron-exon composition shown underneath. Arrows below genes indicate the direction of transcription. Colors correspond to the different histone genes and gene families. Genes flagged with an asterisk (*) represent putative pseudogenes, as inferred from transcriptomic data (see Additional file 1: Fig. S7A, H–J, S8C). Chr: chromosome; Scaff: scaffold. **C** Gene length of the histone variants with inferable orthology across all three annelid taxa, shown as paired data points. **D** Intron length of all introns contained in histone genes. **E**–**G** Gene-wise number of introns (**E**), total intron length (**F**), and average intron length (**G**) in all intron-containing histone genes across all three worm species. Error bars in **D**–**G** are standard deviations. *P* values were derived using one-way ANOVAs, followed by two-tailed post hoc Tukey tests for pair-wise comparisons, wherever applicable. *: *P *value < 0.05; **: *P *value < 0.01; ***: *P* value < 0.001; n.s.: not significant

To investigate the histone gene repertoire of spiralians and annelids, we first reannotated the H2A, H2B, H3, and H4 histone gene models by combining a protein-to-genome alignment-based approach, transcriptomic developmental time series data, a careful manual curation, and an orthology assignment based on maximum likelihood and Bayesian reconstructions (Fig. 2A; Additional file 1: Fig. S1, S2; Additional file 2: Table S1, S2). We recovered 81, 90, and 17 core histone genes for *O. fusiformis*, *C. teleta*, and *D. gyrociliatus*, respectively (Additional file 2: Table S3–S6), belonging to all histone classes, including canonical and non-canonical histones (Fig. 2A). In these annelids, only six, three, and four genes, respectively, displayed an atypical divergent amino acid sequence, which we regard as unknown histone variants. Out of these genes with unknown orthology, all six in *O. fusiformis* and one in *C. teleta* were deemed pseudogenic due to having either null or very low expression levels throughout development. Unlike in other protostomes, where specific histone variants have been lost, like H2A.X in Nematoda [72], or where features and roles from multiple variants get merged into a single gene, like H2A.X and H2A.Z in the H2A.v variant in *Drosophila* [73], all three analyzed annelid taxa have conserved at least one ortholog of each of the core histone proteins—canonical and variant—presumed to be ancestral to Bilateria [14, 74], i.e., the canonical H2A, H2B, H3, and H4 proteins, and the H2A.X, H2A.Z (or H2Av), macroH2A, H3.3, and cenH3 histone variants, suggesting a potential functional conservation of the histone complement. *O wenia* *fusiformis* and *C. teleta* possess between 16 and 23 copies per canonical histone (Additional file 2: Table S3). However, the compact genome of *D. gyrociliatus* only encodes two genes per canonical histone (Additional file 2: Table S3), thus making this fully conserved histone complement one with the lowest (if not the lowest) described copy numbers for canonical histones in a metazoan lineage.

### Genomic organization and gene structure of histones

Canonical histones tend to appear in tandemly repeated, organized clusters, sometimes even associated with H1 linker histones [13, 75, 76]. To assess whether this also occurs in annelids, we characterized the genomic organization of the core histone genes in *O. fusiformis* and *D. gyrociliatus*, the two focal species with more contiguous reference genomes (Fig. 2B; Additional file 1: Fig. S3A). In *O. fusiformis*, canonical core histones are arranged into two large clusters containing 35 and 29 canonical histones in chromosomes 5 and 8, respectively, and a smaller one containing four genes only in chromosome 7. Except for the chromosome 7 cluster and a single occurrence in the chromosome 5 cluster, core canonical histones are tandemly arrayed as an H4–H3–H2A–H2B repeating unit. In this unit, H4, H3, and H2B are transcribed from one strand, and H2A is transcribed from the opposite one (Fig. 2B; Additional file 1: Fig. S3A). Concomitant to the highly reduced histone copy number, the genomic clusters in *D. gyrociliatus* are minimal in size, consisting of a single H3–H4–H2B–H2A repeating unit in scaffolds 2 and 29 (Fig. 2B). The presence of these H2A–H2B and H3–H4 units makes it plausible that previously described head-to-head gene pairs in *C. teleta* and *Helobdella robusta* [74] are also found in *O. fusiformis* and *D. gyrociliatus,* and potentially in other annelids like *Chaetopterus variopedatus* [77]. Interestingly, *O.* *fusiformis* and *D. gyrociliatus* display different genomic organizations than those reported for the annelids *Platynereis dumerilii* (H4–H2B–H2A–H3) [34, 78], *C. variopedatus* (H1–H2B–H3–H4–H2A) [77], and *Urechis capo* (H3–H2A–H2B–H4) [35]. Although these are not based on high-quality reference genome analyses, they indicate that different genomic organizations of the histone complement occur in annelids.

All clustered histone genes lack introns in both species. In contrast, histone variants are often multi-exonic (Fig. 2B; Additional file 1: Fig. S3A). Interestingly, differences in the overall gene length between species are not statistically significant (Fig. 2C; Additional file 1: Fig. S3B, C; Additional file 2: Table S7). However, the overall intron length of histones, as well as the total and average intron length per gene of intron-containing genes, but not the number of introns per gene, are significantly lower in both *C. teleta* and *D. gyrociliatus* compared to *O. fusiformis* (Fig. 2D–G; Additional file 1: Fig. S3D–F). This reduction of average intron length can even be traced gene by gene in the single-copy histone variant genes *h2ax*, *h2az*, *macroh2a*, *cenh3*, and *h3–3* (Additional file 1: Fig. S3G–I). Therefore, annelids organize their histone genes in tandemly repeated clusters. Still, significant changes have occurred in the number of histones, genomic organization, and gene structure throughout annelid diversification.

### Histone genes are in dynamic hyperaccessible chromatin regions

**Fig. 3:**
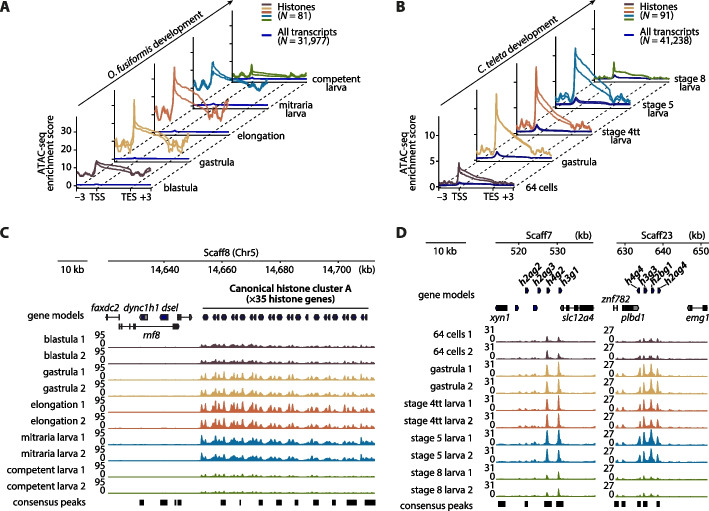
Histone genes are in dynamic hyperaccessible chromatin regions.** A**, **B** ATAC-seq enrichment profiles of histone genes compared to the whole genome during the embryonic development of *O. fusiformis* (**A**) and *C. teleta* (**B**). Distances are in kilobases (kb). TSS: transcription start site; TES: transcription end site. **C** ATAC-seq tracks at one of the two large clusters of canonical histones of *O. fusiformis*, located on chromosome 5. **D** ATAC-seq tracks at two of the six histone gene clusters of *C. teleta* that harbor at least four histones. Note how, in both plots, the ATAC-seq signal is so high for histone genes that the peaks called in neighboring genes are barely visible at the plot scale. *dsel*: dermatan sulfate epimerase like; *dync1h1*: dynein cytoplasmic 1 heavy chain 1; *emg1*: EMG1 N1-specific pseudouridine methyltransferase, *faxdc2*: fatty acid hydroxylase domain containing 2; *plbd1*: phospholipase B domain containing 1; *rnf8*: ring finger protein 8; *slc12a4*: solute carrier family 12 member 4; *xyn1*: xylanase 1; *znf782*: zinc finger protein 782. Chr: chromosome; Scaff: scaffold

To better understand chromatin regulation around histone genes and their genomic clusters, we leveraged our publicly available ATAC-seq developmental time series for *O. fusiformis* and *C. teleta* [66], and data for *D. gyrociliatus* female adults [79] (Additional file 2: Table S8). Histone genes have the highest ATAC-seq enrichment at every developmental point in *O. fusiformis* and *C. teleta*, albeit more starkly in the former (Fig. 3A, B; Additional file 1: Fig. S4A–D). Open chromatin is most prevalent in these species at the transcription start sites (TSS) and promoter regions, progressively decreasing along the gene body towards the transcription end sites. During the embryogenesis of *O. fusiformis* and *C. teleta*, chromatin around histone genes becomes more accessible after the blastula stage during gastrulation, reaching its peak openness and remaining at very elevated levels during larval development (Fig. 3A, B; Additional file 1: Fig. S4A, B). However, chromatin around the histone clusters becomes more compact at the end of the developmental time course. This above-average enrichment is present in the clusters that contain canonical histones—especially in the large clusters of *O. fusiformis*, where it is very significant—and less so in the loci of the variant histone genes (Fig. 3C, D; Additional file 1: Fig. S4C, D, S5A–C, S6A, B). In *D. gyrociliatus*, histone genes are also in loci of highly open chromatin (Additional file 1: Fig. S4E, F). However, their chromatin accessibility landscape differs from those of *O. fusiformis* and *C. teleta*, with chromatin being open almost exclusively at the TSS in defined peaks (Additional file 1: Fig. S5D, S6 C). Therefore, histones are in dynamically regulated, hyperaccessible chromatin regions in annelids, suggesting their expression might be more variable than expected for genes essential for DNA compaction and regulation.

### Histone gene expression dynamics in Annelida

To investigate histone gene expression dynamics across annelid development, we determined all three species’ developmental RNA-seq time courses [66, 79–81] (Additional file 2: Table S9–S15) against the new gene models containing the curated histone genes. Canonical histone gene expression levels were calculated as the sum of the expression of all the genes encoding the same protein (e.g., all 17 genes for caH2A in *O. fusiformis*). All four canonical histones (caH2A, caH2B, caH3, and caH4) present similar expression patterns within species (Additional file 1: Fig. S7A–D, S8A). Moreover, in both species with a larval stage, i.e., *O. fusiformis* and *C. teleta*, histones are expressed in the zygote and cleavage stages—particularly in *O. fusiformis*—but reach maximum expression levels after the zygotic genome activation, at the gastrula stage and immediate post-gastrula stages. They progressively decrease until the competent larva and juvenile stages (Additional file 1: Fig. S7A–C, S8A). This mirrors and correlates with the developmental dynamics of the accessible chromatin regions in which they are located. In the direct developer *D. gyrociliatus*, however, maximum expression of canonical histones occurs at the early embryogenesis time point and plummets immediately after (Additional file 1: Fig. S7A, D, S8A). More interesting are the expression patterns of histone variants, whose expression dynamics across the development of all three studied annelids are conserved, thereby hinting at possible conservation of their roles in development (Additional file 1: Fig. S7A, E–G, S8B). The *h2az* histone, which is known to maintain the pluripotency state of stem cells during development [82, 83], is expressed at high levels in cleavage and early embryogenesis. Its expression decreases after gastrulation when cell fates are acquired, and terminal differentiation into multiple cell lineages starts. The pattern of *h3–3*, which incorporates into chromatin to sustain several differentiation programs across metazoans [84–86], is very similar. The centromeric *cenh3*, critical in kinetochore positioning and assembly during cell division [87, 88], is also highly expressed at cleavage stages when cells divide quickly. Still, its levels dilute earlier and decrease before the blastula forms, unlike *h3–3* and *h2az*. The only case in which expression increases dramatically throughout development to reach a maximum in the adult is the *macroh2a* gene, which may be biologically relevant given its known role in transcriptional repression to maintain the identity of terminally differentiated cells [89, 90]. Given the known relationship between histone gene expression and cell cycle, we adapted a computational method that assigns the cell cycle phase to single cells [91] to our developmental transcriptomes (Additional file 1: Fig. S9). All three species show increasing scores for the G1 phase towards late developmental stages, which matched with the expression pattern of the *macroh2a* gene. Conversely, the expression of *h2az*, *cenh3*, and *h3–3* genes is inversely correlated with the G1 phase dynamics and potentially linked to higher S phase scores in early embryogenesis, particularly in the case of *O. fusiformis*. Nonetheless, proper assessment of the link between cell cycle and histone gene expression dynamics will likely require single-cell resolution approaches. Lastly, we also found distinct expression patterns of the unknown/unidentified histone variants across all species (Additional file 1: Fig. S7A, H–J, S8C), particularly that of *h3uv1* in *O. fusiformis* and *h2buv1* in *C. teleta*, which appear to be highly transcribed genes. We systematically explored the conservation of known hPTM sites in these genes and all other mined histone genes (see Data Availability section), but we still have no hints of their functional relevance.

### Histone H2A.X variants have evolved in parallel in Metazoa

**Fig. 4:**
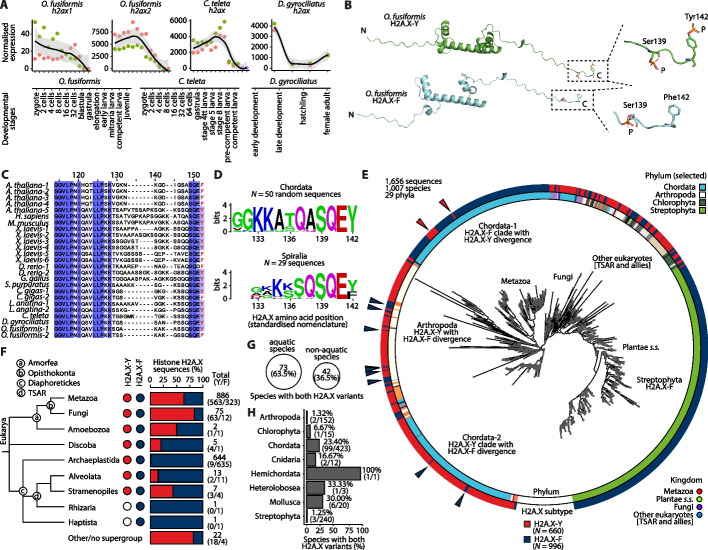
Histone H2A.X variants are a potential source of phenotypic variation in Metazoa. **A** Normalized expression levels of *h2ax* variant histone genes for *O. fusiformis* (left, *h2ax1*; and center left, *h2ax2*), *C. teleta* (center right), and *D. gyrociliatus* (right). Time points are summarized at the bottom for all three RNA-seq time series. Curves are locally estimated scatterplot smoothing, and colored shaded areas represent the standard error of the mean. **B** Renders of AlphaFold3 structural models of the H2A.X-Y and H2A.X-F proteins of *O. fusiformis*. The inset shows a close-up of the C-terminus, where the phosphorylatable Ser139 residues and the Tyr142 and Phe142 residues are depicted as ribbons, highlighting the structural similarities of both amino acids, yet the exclusive phosphorylation of Tyr142. P: phosphate group. **C** Multiple sequence alignment (MSA) depicting the C-terminal region of selected H2A.X proteins, highlighting the variation that can be found in the otherwise conserved Y142 residue (position 152 in the MSA, highlighted in red) in key lineages, particularly in *O. fusiformis* (bottom). *A. thaliana*: *Arabidopsis thaliana*; *H. sapiens*: *Homo sapiens*; *M. musculus*: *Mus musculus*; *X. laevis*: *Xenopus laevis*; *D. rerio*: *Danio rerio*; *G. gallus*: *Gallus gallus*; *S. purpuratus*: *Strongylocentrotus purpuratus*; *C. gigas*: *Crassostrea gigas*; *L. anatina*: *Lingula anatina*. The blue gradient represents the sequence identity for each position in the alignment. For a full-length MSA see Additional file 1: Fig. S11A. (**D**) Sequence logos of the C-terminal region (positions 131–142) of 50 random curated chordate H2A.X sequences (top) and the 29 curated spiralian H2A.X sequences (bottom) obtained from the HistoneDB 2.0 database [104]. (**E**) Maximum likelihood evolutionary reconstruction of PHI-BLAST-retrieved H2A.X-Y and H2A.X-F variants. The phylum of sequences is shown in the inner circle, and the H2A.X subtype/variant is shown in the outer circle, both in a color-coded scale. Colored arrows point to examples of Y-to-F (blue) and F-to-Y (red) conversions within H2A.X-F or H2A.X-Y clades. *s.s.*: sensu stricto; TSAR: Telonemia, Stramenopiles, Alveolata, and Rhizaria. For a fully labelled tree, see Additional file 1: Fig. S12A. **F** Eukaryotic topology as in [105] showing the presence/absence, number, and percentage of the different H2A.X variants in the main major eukaryotic lineages with available data. Circled letters denote key eukaryotic supergroups. **G** Bubble plot proportional to the number and percentage of aquatic and non-aquatic species in the set of species encoding both a H2A.X-Y and a H2A.X-F variant in their genomes. **H** Bar plot showing the number and percentage of species harboring both H2A.X variants in their genomes in the eight phyla that comprised these species

The histone variant H2A.X, critical in DNA repair [92, 93] and embryonic stem cell development [94, 95], among other non-canonical functions [96], is strikingly divergent across the studied annelids. While other variants have a single ortholog in all three species, *C. teleta* and *D. gyrociliatus* display a single *h2ax* gene, but *O. fusiformis* encodes two different H2A.X proteins that share an 88.1% sequence identity (Fig. 2A, B; Additional file 1: Fig. S1, S2, S3A; Additional file 2: Table S3). Both paralogs are maternally deposited in the egg and are highly abundant in early embryonic stages, and progressively lose expression after the blastula and gastrula stages up to their minima in the juvenile adult stage (Fig. 4A; Additional file 1: Fig. S8B). The same expression dynamics are evident for *C. teleta* and *D. gyrociliatus* (Fig. 4A; Additional file 1: Fig. S8B). Nevertheless, while developmental dynamics of both paralogs in *O. fusiformis* are similar, the expression of *h2ax2* is up to 150-fold higher than that of *h2ax1*, confirming that *h2ax2* is the dominant H2A.X gene (Fig. 4A; Additional file 1: Fig. S7A, E). Yet, despite the lower levels of expression, we could confidently prove *h2ax1* is expressed throughout the life cycle of *O. fusiformis* by specifically amplifying both genes from a cDNA pool of mRNA coming from all developmental time points of *O. fusiformis* (Additional file 1: Fig. S10; Additional file 2: Table S16, S17). Remarkably, some of the differences in protein sequence between *h2ax1* and *h2ax2* lie in critical regions, most strikingly in the C-terminal motif of the protein (Fig. 4B, C; Additional file 1: Fig. S11A; Additional file 2: Table S18). In mammals and most chordates, Tyr142 is a conserved position that undergoes biologically relevant posttranslational modifications [97, 98]. Where *h2ax1* displays this phosphorylatable Tyr142 (from here on also H2A.X-Y), *h2ax2* has a sterically homologous yet unmodifiable Phe142 (thus being called H2A.X-F) (Fig. 4B). Indeed, this residue change has been observed before in the H2A.X-F gene of *Xenopus*, where it is expressed in eggs and early embryos and where it has been hypothesized to facilitate rapid early-embryo cell divisions [99, 100]; but also in some fish [99], a mollusc [101], and in plants [99, 102, 103]. Indeed, we found that some of the few curated spiralian H2A.X sequences—and some arthropod and other eukaryote ones—display this change (Fig. 4D; Additional file 1: Fig. S11B–F). Therefore, the distribution of H2A.X-Y and H2A.X-F variants is more widespread than previously foreseen. Yet, it is unknown whether these originated in the last common eukaryotic ancestor or are the result of parallel evolution.

To disentangle the evolutionary history of H2A.X, we performed PHI-BLAST searches for H2A.X-Y and H2A.X-F orthologs across Eukarya combined with phylogenetic reconstruction. We recovered 1656 H2A.X sequences (660 H2A.X-Y and 996 H2A.X-F), mostly belonging to the highly sequenced Metazoa and Plantae clades, across 29 different eukaryotic phyla (Additional file 1: Fig. S12B–G; Additional file 2: Table S19–S23). Few clear trends could be elucidated beyond the fact that almost all plants display an H2A.X-F variant, with the few plant H2A.X-Y variants (nine out of 635) being almost entirely restricted to chlorophytes. The ancestral and most common scenario in arthropods seems to be an H2A.X-Y from which divergent H2A.X-F proteins evolved. Within Chordata, on the other hand, two distinct clades correspond to each of the two H2A.X variants. In each clade, some conversions to the opposite scenario (Y-to-F and F-to-Y transitions) occur; however, there is generally strong conservation of the amino acid at position 142. This robustly suggests that, in chordates, these are different genes with distinct evolutionary origins (Fig. 4E; Additional file 1: Fig. S12A) but that other evolutionary dynamics are also present in animal and other eukaryote lineages (Fig. 4F). Previous research suggested possessing both an H2A.X-Y and an H2A.X-F variant could be an adaptation for quickly developing aquatic species, yet 36.5% of the species with both variants do not display aquatic lifestyles (Fig. 4G; Additional file 2: Table S24). Here we show, however, that species with this two-H2A.X-variant setting are almost exclusively found within Metazoa and are more common than previously thought, as demonstrated in species analyzed from the Chordata (23.40%), Cnidaria (16.67%), and Mollusca (30%) phyla (Fig. 4H), likely reflecting H2A.X-Y and H2A.X-F animal variants to be two distinct genes. These results demonstrate that histone variants can display intricate phylogenetic histories, highlighting the need for further functional comparative analyses of the genome regulatory implications of the two H2A.X variants in eukaryotes and during animal embryogenesis.

### Histone modifiers show a complex evolution throughout annelid diversification

**Fig. 5:**
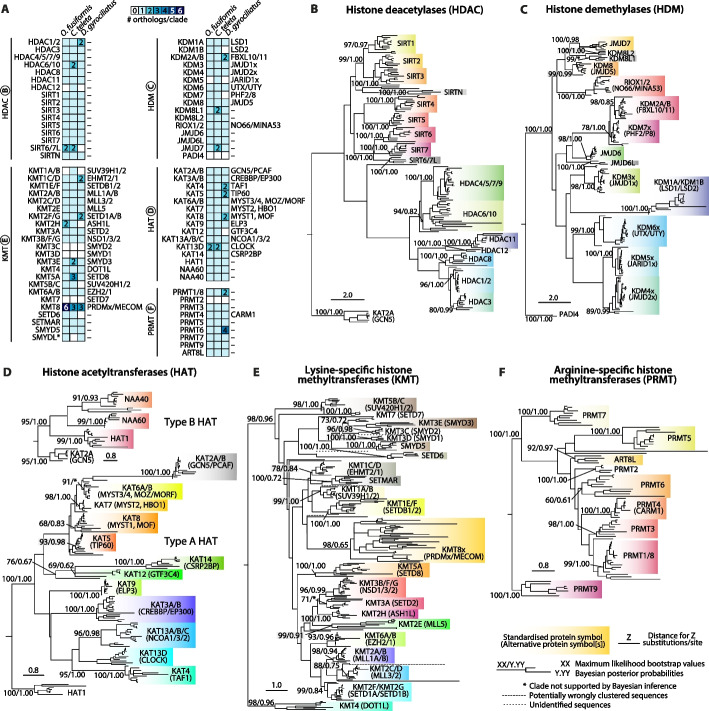
Histone modifiers show a complex evolution throughout annelid diversification.** A** Summary heatmaps of the ortholog number in our annelid taxa of the histone modifiers involved in histone methylation and acetylation: HDAC (top left), HDM (top right), HAT (center right), KMT (bottom left), and PRMT (bottom right). Standardized protein symbols are shown to the left of each summary heatmap, and alternative protein symbols are shown to the right. Some protein symbols are custom for annelid or lineage-specific clades, as described in the text (e.g., SIRT6/7L). Orthologs to more than one gene in mammals are called a single one, separated by strokes (e.g., HDAC1/2). **B–F** Summary phylogenies of the gene orthology assignment of HDAC (**B**), HDM (**D**), type A HAT (**D**, bottom), type B HAT (**D**, top), KMT (**E**), and PRMT (**F**) genes, in *O. fusiformis*, *C. teleta*, and *D. gyrociliatus*. Depicted tree topology is based on the maximum likelihood reconstruction. Branch support values represent bootstrap values (0–100 values) and posterior probabilities (0–1 values) at key nodes. Clades supported by maximum likelihood only are flagged with an asterisk (*). Colored boxes highlight the extent of each histone gene or family. Potentially wrongly clustered sequences and unidentified proteins are shown in long and short-dashed lines, respectively. The scale bar depicts the number of amino acid changes per site along the branches

Besides the histone complement, diversity in the repertoire of histone-modifying enzymes (HME) is the other prominent potential source of evolutionary variation within histone-based regulation. To address this, we investigated the writers and erasers of histone acetylation and methylation: histone deacetylases (HDAC), histone demethylases (HDM), histone methyltransferases (HMT) of both the lysine-specific (KMT) and arginine-specific (PRMT) types, and histone acetyltransferases (HAT), of both type A and type B (Additional file 2: Table S25). We used a mutual best BLAST hit approach to find candidate sequences in the annelid genomes, which were then subjected to orthology assignment using both maximum likelihood and Bayesian reconstructions in six different phylogenetic analyses (Fig. 5; Additional file 1: Fig. S13–S24; Additional file 2: Table S26–S36). We identified a total of 78 clades corresponding to 77 different genes, of which only three (3.9%) were not supported by phylogenetic methods, and only five (6.5%) had bootstrap values or posterior probabilities below 70 or 0.7, respectively, thus making our gene identification highly robust and reliable.

#### Histone deacetylases

Annelids display at least a *hdac1/2*, *hdac3*, *hdac4/5/7/9*, *hdac8*, and *hdac11* ortholog, as well as one ortholog of each of the known NAD-dependent sirtuins, that is, *sirt1*, *sirt2*, *sirt3*, *sirt4*, *sirt5*, *sirt6*, and *sirt7* (Fig. 5A, B; Additional file 1: Fig. S13, S14; Additional file 2: Table S28, S35, S36). There is an additional well-assigned sirtuin clade, which we have here termed *sirtuin novel* (*sirtn*), with a single ortholog in all three species, as well as a clade with high similarity to both *sirt6* and *sirt7* genes (*sirt6/7-like*, or *sirt6/7l*), with two copies present in both *O. fusiformis* and *C. teleta*, but not in *D. gyrociliatus*. The latter presents a duplication of the *hdac1/2* gene, whereas the genome of *C. teleta* encodes for two different *hdac6/10* genes. Intriguingly, *C. teleta* also has an additional HDAC gene that clusters independently, and for which the most similar gene is the poorly characterized *hdac12* gene in zebrafish (Fig. 5A, B; Additional file 1: Fig. S13, S14).

#### Histone demethylases

In terms of histone demethylases, annelids encode orthologs of *kdm1a* (*lsd1*), *kdm1b* (*lsd2*), *kdm2a/b* (*fbxl10/11*), *kdm3* (*jmjd1x*), *kdm4* (*jmjd2x*), *kdm5* (*jarid1x*), *kdm6* (*utx/uty*), *kdm7* (*phf2/8*), *kdm8* (*jmjd5*), *riox1/2* (*no66/mina53*), *jmjd6*, and *jmjd7* (Fig. 5A, C; Additional file 1: Fig. S15, S16; Additional file 2: Table S29, S35, S36). All three species encode a single ortholog of these genes, except for *kdm5*, which is only present in *C. teleta*, the secondary losses of *kdm1b* (*lsd2*) and *kdm7* (*phf2/8*) in *D. gyrociliatus*, and a lineage-specific duplication of *kdm2a/b* (*fbxl10/11*), also in the direct developer *D. gyrociliatus*. We identified a clade with high similarity to *jmjd6*, which we have denoted as *jmjd6-like* (*jmjd6l*), that is nevertheless distinct from *jmjd6* and is present as a single gene in each of the annelids’ genomes. Two other clades phylogenetically closer to *jmjd7* yet automatically assigned as *kdm8* genes were identified. The analyzed genomes contain one copy of each, except in *C. teleta*, which harbors two genes within the *kdm8-like 1* (*kdm8l1*) clade. Similarly to all other invertebrates, the protein arginine deiminase *padi4* is absent in our annelids (Fig. 5A, C; Additional file 1: Fig. S15, S16).

#### Histone acetyltransferases

A complete repertoire of nuclear/type A and cytoplasmic/type B occurs in all three annelids (Fig. 5A, D; Additional file 1: Fig. S17–S20; Additional file 2: Table S30, S35, S36). The only exception is *kat12* (*gtf3c4*), which, despite being part of a general transcription factor (TFIIIC90) of RNA Pol III [106, 107], seems absent in *D. gyrociliatus*. At least one ortholog was found for all three lineages for *kat2a/b* (*gcn5/pcaf*), *kat3a/b* (*crebbp/ep300*), *kat4* (*taf1*), *kat5* (*tip60*), *kat6a/b* (*myst3/4*, *moz/morf*), *kat7* (*myst2, hbo1*), *kat8* (*myst1*, *mof*), *kat9* (*elp3*), *kat13a/b/c* (*ncoa1/3/2*), *kat13d* (*clock*), *kat14* (*csrp2bp*), *hat1*, *naa60*, and *naa40*. Unexpectedly, the compact genome of *D. gyrociliatus* showed the largest expansions, with *kat4* (*taf1*), *kat5* (*tip60*), and *kat8* (*myst1*, *mof*) duplicated in a lineage-specific manner. However, we could also find a likely ancestral duplication common to *O. fusiformis* and *C. teleta* of the *kat13d* (*clock*) gene (Fig. 5A, D; Additional file 1: Fig. S17–S20).

#### Lysine-specific histone methyltransferases

The KMT family showed the most considerable variations between annelids and model organisms. Annelids do not have an ortholog of the chordate-specific *kmt3c* (*smyd2*) and *kmt3d* (*smyd1*) genes [108] or *kmt7* (*setd7*). They do display orthologs for *kmt1c/d* (*ehmt2/1*), *kmt1e/f* (*setdb1/2*), *kmt2a/b* (*mll1a/b*), *kmt2c/d* (*mll3/2*), *kmt2e* (*mll5*), *kmt2f/g* (*setd1a/b*), *kmt2h* (*ash1l*), *kmt3a* (*setd2*), *kmt3b/f/g* (*nsd1/3/2*), *kmt3e* (*smyd3*), *kmt4* (*dot1l*), *kmt5a* (*setd8*), *kmt5b/c* (*suv420h1/2*), *kmt6a/b* (*ezh2/1*), *setd6*, *setmar*, and *smyd5* (Fig. 5A, E; Additional file 1: Fig. S21, S22; Additional file 2: Table S31, S35, S36). Strikingly, we could only assign a *kmt1a/b* (*suv39h1/2*) ortholog in the case of *C. teleta*. *Dimorphilus gyrociliatus* has suffered losses of *kmt2h* (*ash1l*), *kmt5b/c* (*suv420h1/2*), and *setd6*, but carries two different genes of the *kmt1c/d* (*ehmt2/1*) and *kmt2f/g* (*setd1a/b*) classes. *Owenia fusiformis* has a duplication of the *kmt2 h* (*ash1l*) gene, whereas *C. teleta* has one for *kmt3e* (*smyd3*). Moreover, we uncovered an expansion of *kmt5a* (*setd8*) orthologs exclusive of *C. teleta*, which has three different paralogs of this gene. Two sequences of the SET and MYND domain-containing family (SMYD)—one belonging to *O. fusiformis* and another one to *D. gyrociliatus*—did not cluster with any identified KMT clade and were classified broadly as *smyd-like* (*smydl*) genes. Within the *kmt8* (*prdmx/mecom*) clade of genes, both *D. gyrociliatus* and *C. teleta* contained three genes that we could not subclassify but may represent different genes. Surprisingly, *O. fusiformis* displayed a significant expansion in this family, with up to six different genes that could not be resolved further (Fig. 5A, E; Additional file 1: Fig. S21, S22).

#### Arginine-specific histone methyltransferases

Lastly, we also studied the complement of PRMT enzymes (Fig. 5A, F; Additional file 1: Fig. S23, S24; Additional file 2: Table S32, S35, S36). Excluding *prmt2*, which could not be found in any polychaete, all others are present, namely, *prmt1/8*, *prmt3*, *prmt4*, *prmt5*, *prmt6*, *prmt7*, and *prmt9*. A further PRMT gene was identified in all three species that only clustered with the fruit fly *Art8* gene, which we deemed *art8-like* (*art8l*). The expansions of *prmt1/8* and *prmt6* in the genome of *D. gyrociliatus* are thus the only changes within the PRMT family in the studied annelids.

### PRMT6 expansions led to domain fusions and likely catalytically dead enzymes

**Fig. 6:**
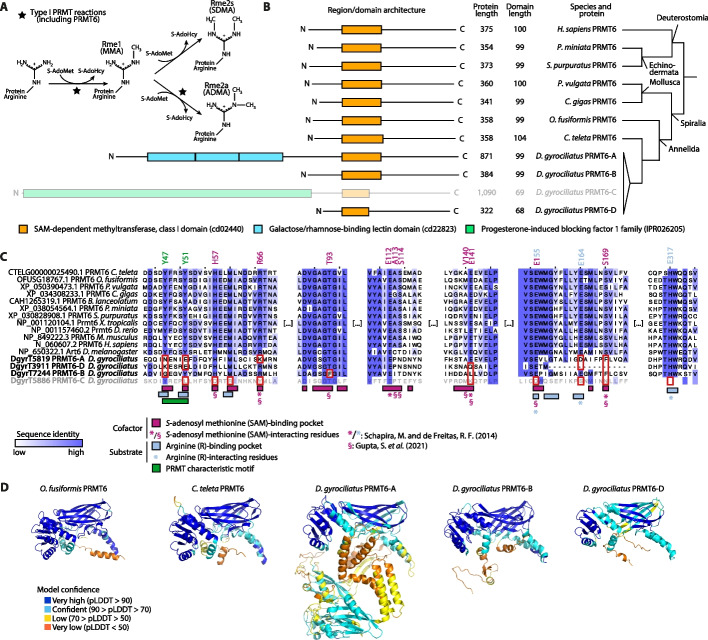
PRMT6 expansions in *D. gyrociliatus *led to domain fusions and likely catalytically dead enzymes.** A** Arginine methylation reactions catalyzed by PRMT proteins. Reactions catalyzed by type I PRMT proteins like PRMT6 are marked with a star. S-AdoMet: *S*-adenosyl methionine/SAM; S-AdoHcy: *S*-adenosyl homocysteine/SAH; Rme1/MMA: monomethyl arginine; Rme2s/SDMA: symmetric dimethyl arginine; Rme2a/ADMA: asymmetric dimethyl arginine. **B** Domain/region architecture of selected PRMT6 proteins. PRMT6-B and PRMT6-D in *D. gyrociliatus* harbor reduced SAM-dependent methyltransferase, class I domains. PRMT6-A and PRMT6-C in *D. gyrociliatus* contain additional domains/regions in the N-terminal region not expected for a PRMT6 ortholog. PRMT6-C may be an annotation artifact and is therefore grayed out. Phylogenetic relationships of the species displayed here are shown to the right of the schematics. *P. miniata*: *Patiria miniata*; *P. vulgata*: *Patella vulgata*. **C** MSA of representative PRMT6 sequences, trimmed to the substrate and cofactor binding regions. At the bottom of the MSA, all four putative PRMT6 orthologs from *D. gyrociliatus* are highlighted in bold. PRMT6-C is grayed out here as well to show its likelihood as an annotation artifact. Key protein regions and residues are highlighted under the MSA. Amino acids with a specified position (as per the human PRMT6 nomenclature) are SAM and arginine-interacting residues. Residues inside red boxes denote conserved positions in key regions or key interacting residues with no conservation in one or more of the orthologs of *D. gyrociliatus*. *X. tropicalis*: *Xenopus tropicalis*. *: SAM-interacting and arginine-interacting residues determined via homology to PRMT4 (CARM1), as in [111]; §: SAM-interacting residues determined directly in PRMT6, as in [112]. For the full-length MSA trimmed to the R43–Y359 positions (as per the human PRMT6 nomenclature), see Additional file 1: Fig. S25. **D** Renders of the AlphaFold3 structural predictions of the annelid PRMT6 orthologs whose gene models were validated using transcriptomics data. From left to right: *O. fusiformis* PRMT6, *C. teleta* PRMT6, and *D. gyrociliatus* PRMT6-A, PRMT6-B, and PRMT6-D. Renders are roughly aligned to the SAM-dependent methyltransferase, class I domain (cd02440). Render color depicts the model’s confidence in the prediction

Considering that *D. gyrociliatus* is a case of extreme genome compaction, frequently associated with gene losses, it is striking that it appeared to possess four different *prmt6* genes (hereon referred to as *prmt6-a* through *prmt6-d*) that subcluster independently of the rest of the sequences of the other species within the *prmt6* clade (Fig. 5A, F; Additional file 1: Fig. S23, S24; Additional file 2: Table S37). PRMT6 enzymes catalyze type II arginine methylation reactions and yield monomethyl arginine (Rme1/MMA) and asymmetric dimethyl arginine (Rme2a/ADMA) (Fig. 6A) in both histones and non-histone proteins and have been shown to regulate cell proliferation and senescence [109, 110]. Both the PRMT6-A and PRMT6-C proteins are significantly longer—with a full length of 871 and 1090 amino acids long, respectively—than what is expected of a PRMT6 ortholog (340–380 residues) (Fig. 6B). We analyzed their domain and region composition and predicted their protein structure (Fig. 6B–D; Additional file 1: Fig. S25, S26) and uncovered that while *O. fusiformis* and *C. teleta* have a very conserved protein structure and domain length, this is only true for *prmt6-b* in *D. gyrociliatus*. Meanwhile, both *prmt6-c* and *prmt6-d* have a shorter *S-*adenosyl methionine (SAM)-dependent methyltransferase class I domain, and *prmt6-a* and *prmt6-c* contain additional regions and domains in the N-terminal region of the protein, namely three consecutive galactose/rhamnose-binding lectin domains and a progesterone-induced blocking factor 1 family region, respectively (Fig. 6B, D; Additional file 1: Fig. S26A). Transcriptomic validation of the fusion genes showed that the one present in *prmt6-c* is, however, likely a false positive and a result of a wrongful annotation. Unlike in *prmt6-a*, where both presupposed fused parts of the gene show continuous transcription at similar levels, the *prm6-c* gene showed discrepancies in read density across the model, with many unexplained antisense reads (Additional file 1: Fig. S26B, C). No PRMT6 ortholog contains the functionally critical tyrosine dyad (Y47 and Y51) in the PRMT characteristic motif (Fig. 6C; Additional file 1: Fig. S25). There are also a very high number of amino acid changes in highly conserved positions known to interact with either the SAM cofactor (e.g., R66, E141, and S169) and/or with the arginine residue (e.g., E164 and E317) (Fig. 6C; Additional file 1: Fig. S25). In fact, structural changes affecting the characteristic PRMT domain are evident, most notably in the lack of tertiary structures of the alpha helices where the PRMT characteristic motif and the tyrosine dyad are located (Fig. 6D; Additional file 1: Fig. S26A). These sequence and structure alterations are likely disrupting the enzymatic function potential of PRMT6, thus perhaps relaxing selection pressures and allowing for various gene expansions and divergence events to occur. Altogether, our data indicate that in an annelid with a highly compact genome and a reduced and simplified histone complement, like *D. gyrociliatus*, the plasticity in histone-based regulation required during embryogenesis most likely resides in the conservation and, in some cases, diversification of the HME repertoire.

### Life cycle correlates with heterochronies of histone modifiers

**Fig. 7:**
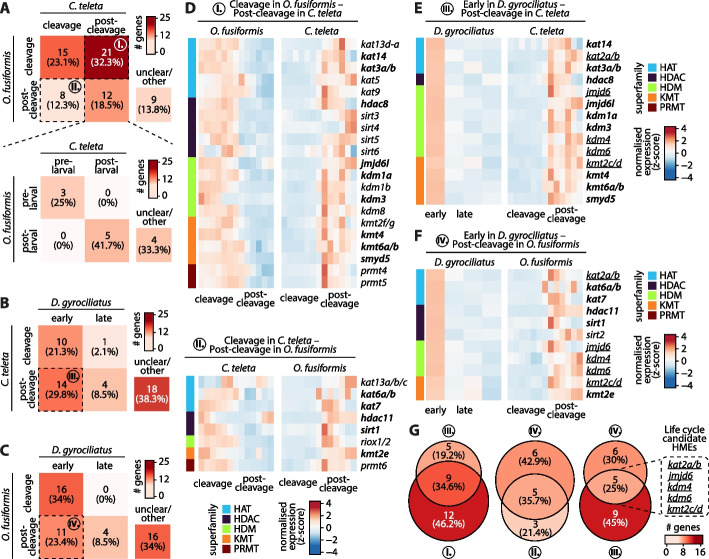
Life cycle correlates with heterochronies of histone modifiers. **A**–**C** Pairwise comparative gene expression analyses of HMEs across species by classifying clusters of temporally co-regulated genes into early/pre-cleavage and late/post-cleavage clusters and pre- and post-larval clusters. The color scale denotes the number of genes. Species compared are *O. fusiformis* and *C. teleta* (**A**), both using the cleavage/post-cleavage classification (**A**, top) and the pre- and post-larval one (**A**, bottom); *C. teleta* and *D. gyrociliatus* (**B**); and *O. fusiformis* and *D. gyrociliatus* (**C**). Unclear/other includes comparisons of genes clustered in an unclassified transitional cluster in at least one of the two species (see Additional file 1: Fig. S27). Dotted lines highlight the gene sets (I–IV) of genes under heterochronic shifts. **D**–**F** Heatmaps of normalized expression dynamics of the gene sets of genes under heterochronic shifts. Color scale denotes normalized gene expression in a *z*-score scale. Species compared are *O. fusiformis* (left) and *C. teleta* (right) (**D**, top, gene set I); *C. teleta* (left) and *O. fusiformis* (right) (**D**, bottom, gene set II); *D. gyrociliatus* (left) and *C. teleta* (right) (**E**, gene set III); and *D. gyrociliatus* (left) and *O. fusiformis* (right) (**F**, gene set IV). **G** Venn diagrams of the intersections of heterochronic gene sets. The color scale denotes the number of genes. Intersections between gene sets I and III (left) and gene sets II and IV (center) represent species-specific heterochronies related to larval development in *O. fusiformis* and *C. teleta*, respectively, which correspond to gene symbols in bold in **D**–**F**. Intersection between gene sets III and IV (right) represent consistent heterochronies related to the life cycle, corresponding to gene underlined gene symbols in **E** and **F**

Histone-modifying enzymes (HMEs) constitute a highly plastic source of regulatory variation that underpins changes in gene expression patterns [9, 21, 113–115]. To investigate how temporal shifts in deploying orthologous developmental programs could correlate with changes in HME expression, we leveraged the entire developmental RNA-seq time series, from the zygote to competent larva/adult stages, for all three annelid species. All expressed transcripts were clustered using an unbiased soft *k*-means approach into an optimal number of 15 clusters (*O. fusiformis* and *C. teleta*) or eight clusters (*D. gyrociliatus*) (see Methods, Additional file 1: Fig. S27A–C; Additional file 2: Table S38–S40). Next, we assigned the clusters to their corresponding developmental stages according to their maxima and classified them as cleavage or post-cleavage clusters (*O. fusiformis* and *C. teleta*) or early development and late development clusters (*D. gyrociliatus*). We further subdivided post-cleavage clusters in the indirect developers into pre-larval and post-larval clusters (Additional file 1: Fig. S27D–F). We profiled the expression dynamics of all five superfamilies of histone modifiers described above across the development of the three species. Given the broad array of biological readouts, different modifiers from the same superfamily can have, as expected, no clear superfamily-specific trends common to all three taxa (Additional file 1: Fig. S28, S29; Additional file 2: Table S41–S43). We then decided to focus on gene-level expression dynamics. To do this, we compared the onset of expression of every histone modifier with single-copy orthology either between species with indirect development or common to all three species (Fig. 7; Additional file 1: Fig. S30, S31; Additional file 2: Table S44, S45). When comparing *O. fusiformis* and *C. teleta*, we unravelled more histone modifiers under heterochronic shifts between cleavage and post-cleavage clusters (29 genes, 44.6% of the total) than sharing the same expression pattern (27 genes, 41.5%). This is primarily because 21 enzymes (32.3%) are expressed early during cleavage stages in *O. fusiformis* but late in post-cleavage time points in *C. teleta*. Yet, the opposite is also true for eight enzymes (12.3%) (Fig. 7A, top). Heterochronic genes belong to all five superfamilies of modifiers. In the first group of genes (group I), or those delayed in the lecithotrophic larva, we can find, among others, the H3K27ac acetyltransferase *kat3a/b* (*crebbp/ep300*) and one of the circadian clock gene orthologs *kat13d-a* (*clock-a*); four different sirtuin genes, namely *sirt3*, *sirt4*, *sirt5*, and *sirt6*; both lysine-specific demethylases *kdm1a* (*lsd1*) and *kdm1b* (*lsd2*); the H3K79-specific *kmt4* (*dot1l*) and H3K27-specific *kat6a/b* (*ezh2/1*); as well as *prmt4* and *prmt5*. Displaced towards a late expression in the planktotrophic larva (group II), examples include the ortholog to the steroid receptor co-activators *kat13a/b/c* (*ncoa1/3/2*),* hdac11*, and *sirt1*, the bifunctional histone demethylase/ribosome hydroxylase *riox1/2* (*no66/mina53*), the inactive *kmt2e* (*mll5*), and the previously discussed *prmt6* gene (Fig. 7A, top, D; Additional file 1: Fig. S30). Contrary to our null expectation of finding heterochronic shifts between pre-larval and post-larval expression, all genes with a post-cleavage expression were consistently deployed at the same life cycle time point—either before or after larval formation—between *C. teleta* and *O. fusiformis* (Fig. 7A, bottom), suggesting that histone-based regulatory mechanisms in later developmental stages might be broadly conserved between indirect developing annelids.

We thus focused on comparing direct and indirect developers, i.e., *D. gyrociliatus* and *C. teleta* or *O. fusiformis*. As observed with transcription factors [66], a significant number of HMEs are pre-displaced from late/post-cleavage expression in *C. teleta* (group III, 14 genes, 29.8%) and *O. fusiformis* (group IV, 11 genes, 23.4%) to early development in *D. gyrociliatus* (Fig. 7B, C). Genes involved in these shifts are varied, with all superfamilies except the PRMT enzymes being represented in both groups of heterochronic genes (Fig. 7E, F; Additional file 1: Fig. S31). A critical advantage of these pairwise comparisons is that we can intersect them and find those changes that are consistent and specific to direct and indirect developers (Fig. 7G). By comparing groups I and III, those where genes are shifted from early/cleavage expression in either *O. fusiformis* or *D. gyrociliatus* to late/post-cleavage expression in *C. teleta*, we can robustly describe modifiers specific to the development of the lecithotrophic larva of *C. teleta* (Fig. 7D, top, E, G, left). These are *kat3a/b* (*crebbp/ep300*), *kat14* (*csrp2bp*), *hdac8*, *jmjd6l*, *kdm1a* (*lsd1*), *kdm3* (*jmjd1x*), *kmt4* (*dot1l*), *kmt6a/b* (*ezh2/1*), and *smyd5*. The equivalent can be produced for *O. fusiformis* by comparing groups II and IV, which yields the modifiers specific to the development of the planktotrophic larva of *O. fusiformis* (Fig. 7D, bottom, F, G, center), namely *kat6a/b* (*myst3/4*, *moz/morf*), *kat7* (*myst2*, *hbo1*), *hdac11*, *sirt1*, and *kmt2e* (*mll5*). Lastly, we compared groups III and IV, i.e., those genes that are consistently pre-displaced to early expression in *D. gyrociliatus* from late/post-cleavage expression in both indirect developers (Fig. 7E, F, G, right), which included *kat2a/b* (*gcn5/pcaf*), three different demethylases, *jmjd6*, *kdm4* (*jmjd2x*), and *kdm6* (*utx/uty*), and *kmt2c/d* (*ehmt2/1*). Therefore, a few orthologous HMEs exhibit different temporal expression dynamics between the studied annelids. These might represent key epigenetic regulators of specific developmental programs that differ between annelids with direct and indirect development, thus emerging as a tractable gene set for future functional manipulations.

### Adult annelids harbor distinct histone modification enrichments

**Fig. 8:**
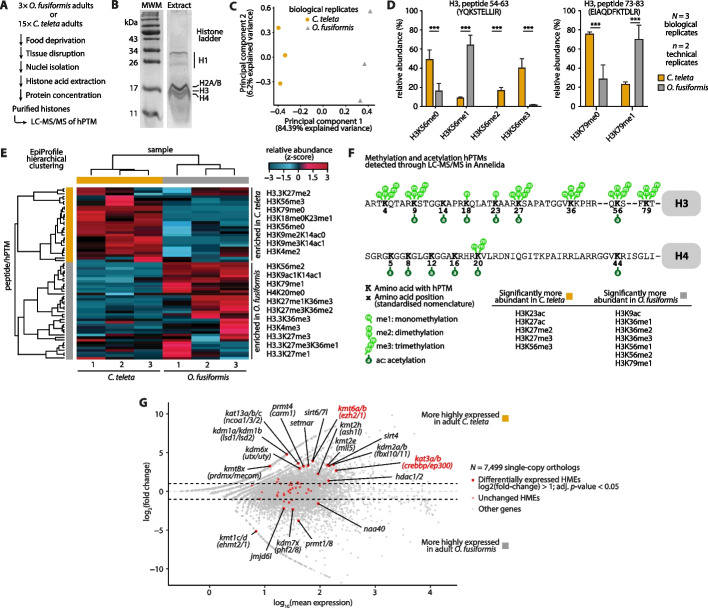
Adult annelids harbor distinct histone modification enrichments.** A** Histone acid extraction protocol schematic. **B** Sample SDS-PAGE analysis of acid-extracted histones from 15 adult specimens of *C. teleta*. See uncropped gel in Additional file 1: Fig. S32B. **C** Principal component analysis of the histone H3 and H4 hPTM profiles derived from LC-MS/MS experiments, averaged by biological replicate, for all analyzed samples of *O. fusiformis* and *C. teleta*. **D** Relative abundance bar plots of the H3 54–63 peptide (YQKSTELLIR) based on H3K56 methylation status (left), and the H3 73–83 peptide (EIAQDFKTDLR) based on H3K79 methylation status (right), in *O. fusiformis* and *C. teleta*. Error bars represent standard deviation. *P* values were derived from two-tailed Student’s *t* tests. ***: *P* value < 0.001. **E** Hierarchically clustered heatmap of the relative abundance of all detected histone H3 and H4 peptide/hPTMs. The numbers below samples are for biological replicates. In orange, peptides/hPTMs enriched in *C. teleta* samples; in gray, those enriched in *O. fusiformis*. The list of peptides/hPTMs to the right of the heatmap only includes representative examples. **F** Annelid acetylation and methylation hPTM landscape in histones H3 and H4. hPTMs that are statistically significantly more abundant in either species are listed here, based on quantifications shown in **D** and Additional file 1: Fig. S32E–H. **G** MA plot depicting the pairwise comparison of adult RNA-seq datasets from *C. teleta* and *O. fusiformis*. Genes with positive log_2_(fold-change) values have higher expression values in *C. teleta*, while negative values indicate higher expression in *O. fusiformis*. Red dots highlight the position of the HMEs with single-copy orthology, of which those with a larger dot size are differentially expressed across the two species

Given the differences in the HME repertoires between species and their expression timing across development, we investigated whether adults may display different levels of key histone modifications. To assess this, we used quantitative mass spectrometry-based detection of hPTMs in adult specimens of *O. fusiformis* and *C. teleta* and focused on the methylation and acetylation of residues in H3 and H4 histones (Fig. 8A, B; Additional file 1: Fig. S32A–C; Additional file 2: Table S46, S47). The three biological replicates with two technical replicates strongly clustered by species (Fig. 8C; Additional file 1: Fig. S32D). Significant differences in key posttranslational modifications (hPTMs) were immediately evident in several peptides. Notably, the most prominent differences were observed in H3K56 methylation and H3K79 methylation. H3K56 is mostly unmethylated (H3K56me0) or di- and trimethylated (H3K56me2 and H3K56me3) in *C. teleta* but more abundant in its monomethylated form (H3K56me1) in *O. fusiformis* (Fig. 8D, left). H3K56me3 is known to be introduced in humans by the KMT1A (SUV39H1) and KMT1B (SUV39H2) enzymes. The almost exclusive presence of H3K56me3 in *C. teleta* matches our predictions, given that the annelid KMT1A/B (SUV39H1/2) ortholog is present in *C. teleta* but absent in *O. fusiformis*. On the contrary, the ortholog to the human H3K56me1-depositing enzyme KMT1D (EHMT2), which is KMT1C/D (EHMT2/1) in annelids, is present in *O. fusiformis*, likely explaining the accumulation of H3K56me1 in this species. The functional and biological implications of these differences are not obvious, as these hPTMs are not extensively characterized in model systems. H3K56me1 is involved in DNA replication regulation [116], while H3K56me3 is a heterochromatic mark that largely overlaps with the constitutive heterochromatin mark H3K9me3, especially in the pericentromeric regions [117, 118]. However, it might be that the different species-specific levels of H3K56 modifications are a non-adaptive direct consequence of the variations in the presence/absence and expression of HMEs regulating these histone marks.

We also found significant differences in unmodified and monomethylated H3K79, with the former being the dominant form in *C. teleta* and the latter in *O. fusiformis* (Fig. 8D, right). Interestingly, H3K79me1 has been linked to alternative splicing and transcription in cell lines and *C. elegans* [119–121]. Some well-studied and conserved methylation hPTMs like H3K4me2 and H3K4me3—among many others—appeared to be more enriched in one species from EpiProfile analyses, though statistical quantifications showed no significant differences between species (Fig. 8E; Additional file 1: Fig. S32E). Other methyl marks like H3K36me1/2/3 and H3K27me2/3 were significantly more abundant in one of the annelid lineages. In the case of H3K27me3, a transcriptional repressive mark often found in developmentally regulated bivalent promoters [122], the higher abundance in *C. teleta* adults correlates well with the heterochronic shift between early/cleavage expression in *O. fusiformis* and late/post-cleavage expression in *C. teleta* of *kmt6a/b* (*ezh2/1*), the catalytic subunit of the Polycomb repressive complex 2 (PRC2). We also found this to be true for two acetylations, namely H3K23ac and H3K27ac, both significantly more abundant in *C. teleta* (Fig. 8F; Additional file 1: Fig. S32F–H), which could be the result of the same type of heterochronic shift between early/cleavage expression in *O. fusiformis* and late/post-cleavage expression in *C. teleta* of five different HAT genes: *kat5* (*tip60*), *kat9* (*elp3*), *kat13d-a* (*clock-a*), *kat14* (*csrp2bp*), but most interestingly, *kat3a/b* (*crebbp/ep300*). H3K9ac, however, escapes this pattern and appears more abundant in *O. fusiformis* (Additional file 1: Fig. S32F). Likewise, this may be associated with chromatin and transcription differences in the annelids, as histone acetylations, such as H3K23ac and H3K27ac, are known to be tightly linked to increased transcription across species [123–125]. Therefore, adult annelids exhibit significant differences in the levels of hPTMs associated, in model systems, with transcriptional regulation. Although the exact biological implications of these in annelids are uncertain without functional investigations, they may perhaps reflect age-associated differences in genome regulation, as also observed with DNA methylation [126].

Finally, we complemented the mass spectrometry approach with an analysis of adult RNA-seq datasets of *C. teleta* and *O. fusiformis* [126], in which we focused on the differential transcript abundances of the HMEs (Fig. 8G; Additional file 1: Fig. S33; Additional file 2: Table S48, S49). Several of them were differentially expressed across species. Most excitingly, however, was the much higher expression in *C. teleta* adults of *kat3a/b* (*crebbp/ep300*) and *kmt6a/b* (*ezh2/1*), in agreement with their heterochronic shift during development, as well as the differential abundance of the hPTMs they introduce, that is, H3K27ac and H3K27me3, respectively. Altogether, our data describe a core repertoire of annelid histone methylations and acetylations of histones H3 and H4, revealing some key differences in the adult levels of key hPTM, likely the result of differences in the repertoire and expression dynamics of HMEs. Future gene-specific functional studies and nucleosome-level resolution hPTM profiling will clarify the role of these epigenetic modifications and the biological implications of their differential usage in the adults of *O. fusiformis* and *C. teleta*.

## Discussion

In this study, we profiled the histone, HME, and hPTM repertoires to trace the evolution and developmental dynamics of histone-based regulation in three annelid lineages with contrasting genomic features and ontogenetic trajectories (Fig. 1B). Through a multi-omics approach combining comparative genomics, public bulk RNA-seq and ATAC-seq datasets, and the generation of mass spectrometry proteomic data, we identified multiple layers of histone-based regulation common and specific to these annelids that, in some cases, correlate with their life cycles and developmental modes, raising testable and tractable hypotheses to explore further how this genome regulatory mechanism underpins animal embryonic and life cycle evolution.

Our data underscore the extremely plastic evolutionary dynamics of animal histone numbers and genomic organization. Our orthology analysis, using a limited taxon sampling of well-annotated histone repertoires and rooted based on the linker histone H1, retrieved all histone classes, including canonical and non-canonical histones, as strongly supported monophyletic clades (Fig. 2A). However, this approach did not retrieve the more probable phylogenetic relationships between core canonical histones as proposed in a recent, more comprehensive study that analyzed archaeal, viral, and eukaryotic sequences, proposing two rounds of gene duplication and the one-to-one paralogy between H2A–H3 and H2B–H4 in the evolution of the nucleosomal histones [3]. Remarkably, *D. gyrociliatus* displays one of the lowest (if not the lowest) characterized histone copy number in a free-living animal but an essentially complete (and expanded in some instances) HME repertoire. While we cannot exclude the possibility that the latter may be explained by the diverse range of non-histone targets of HMEs, paradoxically, this setting is opposite to that of *Oikopleura dioica*, another animal lineage with a highly compact genome that has, however, experienced rapid innovation in its histone repertoire [127]. Therefore, genome reduction impacts histone evolution in different ways. More importantly, our study reveals *D. gyrociliatus*, with its reduced histone repertoire, as an ideal system to investigate the functional impact of hPTMs in animals, as it would simplify the more complex functional investigations implemented in other organisms with more redundant histone repertoires, such as *D. melanogaster* [128, 129] and, more recently, mice [130].

All five studied annelid species exhibit different arrangements of the repeating unit in their histone clusters, which can be explained by lineage-specific genomic translocations from an undetermined ancestral state. The genomic organization of histone genes of *O. fusiformis* and *D. gyrociliatus* determined in this study revealed that H3 and H4 genes are contiguous and always transcribed from the same strand. Yet, when compared with other annelids and molluscs [77, 131, 132], multiple chromosomal rearrangements are required to explain the genomic organization differences. Interestingly, *C. variopedatus* exhibits a repeated histone quintet that includes the histone linker H1 [77], a feature also observed in arthropods, echinoderms, molluscs (bivalves, but not gastropods), chordates, and the cnidarian *Hydractinia* (but not the coral *Acropora*), although the order of histones differs in each case [77, 133, 134]. It is thus plausible that this quintuple, as observed in *Hydractinia* (H2A–H2B–H4–H3–H1) or even sea urchins (H1–H4–H2B–H3–H2A, all in the same transcriptional orientation and matching the possible phylogenetic relationships of core canonical histones), represent the ancestral state for Bilateria. This repeating unit would have experienced lineage-specific expansions, rearrangements (e.g., to favor head-to-head gene pairs to increase transcriptional efficiency), and the frequent loss of the linker histone H1 during bilaterian diversification. However, the relationship between these different genomic organizations and other aspects of chromatin and gene regulation is unclear. Many gene regulatory mechanisms are conserved between vertebrates, *D. melanogaster* and *C. elegans*, the main research model systems, despite their differences in histone cluster organization and histone complement. Interestingly, however, there appears to be a correlation between the erosion of ancestral linkage groups and potential divergence in the organization of the core histone clusters, as *O. fusiformis* and *D. gyrociliatus* exhibit more rearranged genomes than echiurids and nereids [66, 79, 135]. Nonetheless, a deeper taxon sampling exploiting the increasing number of chromosome-level assemblies for most animal phyla and their closest outgroups is essential to resolve the most probable evolutionary dynamics of histone gene organization.

Despite some differences in expansions and losses, the complements of HMEs are generally conserved in and between annelids (Fig. 5A), in sharp contrast to the substantial differences we recently described in the DNA methylation levels and associated machinery for these same species [126]. Accordingly, some of these differences affect HMEs that interact with the DNA methylation machinery in mammals [136–138]. These three annelids exhibit varying DNA methylation levels and complements of writers and erasers [126]. In particular, *D. gyrociliatus* has negligible levels of 5-methylcytosine, lacks the de novo DNA methyltransferase DNMT3, and exhibits divergent orthologs for DNMT1 and UHRF1 [126]. Interestingly, this annelid also lacks KMT1A/B (SUV39H1/2) and has a duplication of KMT1C/D (EHMT2/1), which in mammals recruit the DNA methylation machinery to H3K9me3 heterochromatic regions [139, 140]. Notably, among the three annelids, only *C. teleta* exhibits a KMT1A/B ortholog (Fig. 5A, E). In mammals, this protein interacts directly with the DNA methylation reader MeCP2 [141, 142], and *C. teleta* is the only of the three annelids with an MBD-containing ortholog that could functionally link DNA methylation with hPTMs, suggesting that this interaction might be conserved, or at least possible, in this worm. In addition, *D. gyrociliatus* lacks the histone H3K4 demethylase KDM1B (LSD2), which is required for proper de novo DNA methylation of particular imprinted loci during mammalian oogenesis [143]. Finally, annelids and other invertebrates lack a *padi4* ortholog, which citrullinates DNMT3, stabilizing this DNA methylation writer and promoting the deposition of methyl-groups [144]. It is thus tempting to speculate that the evolution of this interaction contributed, at least partially, to establishing the hypermethylation state of vertebrate genomes.

While previous work recapitulating molecular heterochrony invoked several epigenetic regulators, most notably microRNAs (miRNAs) [145–149], less is known about the role of the histone-based regulation machinery in this fundamental developmental and evolutionary process. Through comparative gene expression analyses of HMEs, we discovered that the expression dynamics of HMEs are not always conserved between annelids. Instead, in some cases, more than 50% of the studied genes are pre- or post-displaced between larval phenotypes and between species possessing or lacking a larval stage. Interestingly, HDMs were the most popular category of HMEs that consistently shifted between late expression in indirect developers and early expression in *D. gyrociliatus*, the same heterochrony type previously described for the trunk patterning program [66]. These included *kdm4* (*jmjd2x*), *kdm6* (*utx/uty*) and *jmjd6*, whose known roles in mammals [150–154] might indicate a potential removal of methylation marks of both permissive (H3K36me3) and repressive nature (H3K27me3 and H3K9me3) earlier in direct development to accelerate the expression and subduing of pre-displaced transcriptional programs. Nonetheless, a prior characterization of the genetic and cellular underpinnings of the traits subject to heterochronic shift, such as in the formation of the trunk, will be essential to discriminate a causal role of changes in hPTMs and HME expression from an indirect consequence of the different dynamics in the unfolding of developmental programs in annelids with distinct lifestyles.

## Conclusions

Altogether, our work highlights the power of and the need to expand the study of histones and histone-based regulation to non-model systems to attain a more comprehensive understanding of the evolutionary dynamics shaping a core layer of genome and gene expression regulation. Recent technical advances, such as CUT&RUN and CUT&Tag, enable nucleosome-level resolution hPTM profiling from limited input material [39, 42]. This, along with the increase in high-quality chromosome-level assemblies, should overcome the traditional barriers to studying histone-based regulation in non-model systems. In this regard, our work provides the analytical means to expand the characterization of the histone-based regulatory complex to emerging, previously uncharacterised taxa. Furthermore, the comparison of annelids with different genomic and developmental traits defined a tractable set of HMEs and hPTMs correlated with phenotypic differences whose detailed study through histone profiling and, most importantly, loss-of-function studies (e.g., CRISPR-Cas9) will help understand how variation in chromatin regulation contributes to the fascinating diversification of annelids. Ultimately, this will help identify shared and divergent features in the evolution of chromatin regulation in animals and eukaryotes.

## Methods

### Adult culture

Sexually mature *Owenia fusiformis* Delle Chiaje, 1844 adults were obtained from subtidal waters near the Station Biologique de Roscoff (Centre National de la Recherche Scientifique (CNRS)-Sorbonne University, Roscoff, France) and cultured in-house as described before [71]. *Capitella teleta* Blake, Grassle & Eckelbarger, 2009 and *Dimorphilus gyrociliatus* (O. Schmidt, 1857) were cultured following previously described protocols [70, 155].

### Histone genes mining

Human core histone protein sequences, i.e., H2A, H2B, H3, and H4 proteins, were retrieved from NCBI following the gene definitions provided in the HUGO Gene Nomenclature Committee (HGNC). Curated histone variants sequences from model organisms corresponding to the H2A.X, H2A.Z/H2Av, H3.3, cenH3/CENP-A, and macroH2A subfamilies were downloaded from the NCBI Histone Variants Database 2.0 (HistoneDB 2.0) (https://www.ncbi.nlm.nih.gov/research/histonedb) [104] (Additional file 2: Table S1). The protein2 genome model of the exonerate tool (EMBL-EBI) was employed to align all fetched sequences to either the unmasked or softmasked scaffold-level genome assemblies of *O. fusiformis* [66], *C. teleta* [156], and *D. gyrociliatus* [79]. The resulting gff files containing putative orthologs were formatted to gff3 format using custom code. The AGAT v.0.6.0. suite of scripts [157] and the gffutils v.0.8.4 package (https://github.com/daler/gffutils) were used to merge transcripts with overlapping codifying sequences, remove identical transcripts, and keep the longest isoform of gene models containing monoexonic transcripts only. Resulting putative sequences were aligned by histone family using MAFFT v.7. [158] with the G-INS-i iterative refinement method, BLOSUM62 as a scoring matrix and a 1.53 gap penalty. Multiple sequence alignments (MSAs) were used as support, together with visual inspections of the corresponding genomic loci in IGV v.2.8.13. [159], to manually curate histone sequences. Transcripts around or in poorly sequenced regions of the genomes and those with non-canonical splicing sites were discarded. Sequences with frameshifts or in-frame termination codons were also discarded. The sequence of multiexonic transcripts was inferred from full-length RNA-seq transcripts from previously published data for all three species [66, 79, 80] found through a tblastn search [160] of partial alignments. Only a single transcript per gene and locus was kept, as alternative splicing leading to different protein sequences is rare in histone genes [161, 162] and could not be inferred with certainty from a genome alignment-based approach. Gene models were then lifted off from the scaffold-level assembly of *O. fusiformis* (GenBank: GCA_903813345.1) to the updated chromosome-level assembly (GenBank: GCA_903813345.2) using Liftoff v.1.6.1 [163]. Curated protein sequences were then extracted using gffread v.0.12.1 [164]. Alignments and the corresponding alignment files in FASTA and Nexus formats, updated filtered gene models, transcript and protein files, and genome annotation reports for all three annelid taxa are available in the GitHub repository and Zenodo (see Data availability section).

### Histone genes orthology assignment

Curated core histone and histone variant sequences from model organisms were retrieved from the NCBI (Additional file 2: Table S2) and aligned with the curated annelid histones. Sequences were aligned using MAFFT v.7. [158] as described above. To validate a preliminary sequence-based orthology assignment, a phylogenetic analysis was performed on the resulting MSA with a rtREV amino acid substitution matrix [165] to account for transition rates. We allowed for a proportion of invariable sites (+ I) and used the discrete gamma model [166] with four rate categories (G4) to describe site evolution rates, together with an optimization of amino acid frequencies using maximum likelihood (ML) in IQ-TREE v.2.0.3 [167]. One thousand ultrafast bootstraps (BS) [168] were used to extract branch support values, but bootstrapping convergence was reached earlier. Further support in the form of posterior probabilities was obtained from Bayesian reconstruction in MrBayes v.3.2.7a [169], using the same rtREV + F + I + G4 model. Two runs with four chains of Markov chain Monte Carlo (MCMC) analyses were run for 50,000,000 generations. Tree visualization and editing were done using FigTree v.1.4.4 (http://tree.bio.ed.ac.uk/software/figtree/). The resulting histone gene orthology assignment is summarized in Additional file 2: Table S3 and described in detail for each species in Additional file 2: Table S4–S6. Alignments and the corresponding alignment files in FASTA and Nexus formats are available in the GitHub repository and Zenodo (see Data availability section).

### Histone gene structure analysis

Histone gene structure was analyzed using the GenomicFeatures v.1.46.5 and GenomicRanges v.1.46.1 packages [170]. Gene length, aggregated intron length, total and average intron length/gene, and total and average intron number/gene were analyzed. For gene structure comparisons between the three species of the histone variants with known role and single-copy orthology, i.e., H2A.Z, H2A.X, macroH2A, cenH3, and H3.3, repeated measures one-way ANOVAs were performed, followed by two-tailed paired Student’s *t* test, when applicable. For *O. fusiformis*, we considered the *h2ax2* gene (H2A.X-F variant) the H2A.X ortholog, as it is the paralog with the highest expression levels in every developmental stage. For all other comparisons, we employed one-way ANOVAs, followed by two-tailed post hoc Tukey tests for pair-wise comparisons, when applicable. All *P* values derived from pair-wise comparisons were adjusted using the stringent Bonferroni method for multiple testing correction (Additional file 2: Table S7).

### Chromatin accessibility profiling

Chromatin accessibility dynamics were analyzed using publicly available coverage.bw files corresponding to ATAC-seq experiments for all three species [66, 79] (Additional file 2: Table S8). Metagene enrichment analysis was computed using the computeMatrix and plotHeatmap commands in deepTools v.3.4.3 [171]. Peak tracks and gene structures were visualized using pyGenomeTracks v.2.1 [172] and deepTools v.3.4.3 [171].

### Developmental gene expression profiling

Gene expression dynamics were re-profiled using publicly available developmental transcriptomes for all three species [66, 79–81] (Additional file 2: Table S9) to include the previously incomplete histone gene models, from zygote to juvenile stages for *O. fusiformis*, from zygote to competent larva stages for *C. teleta*, and from early embryogenesis to female adult for *D. gyrociliatus*, as described before. Trimmomatic v.0.3980 [173] was used to remove sequencing adaptors. Clean reads were pseudo-aligned to the updated filtered gene models using kallisto v.0.46.2114 [174], and DESeq2 v.1.30.1 [175] was used to normalize counts between samples within each species (Additional file 2: Table S10–S15). Unidentified variant genes with DESeq2 normalized expression values below ten for all developmental time points and biological replicates were flagged as putative pseudogenes (Additional file 2: Table S4–S6). Gene expression matrices in TPM and DESeq2 normalized values for all three species are available in the GitHub repository and Zenodo (see Data availability section). We repurposed the cyclone “marker pairs” approach for computationally predicting the cell cycle phase [91] to the developmental transcriptomes. The assignment was carried out using the cyclone function of the scran v1.32.0 package with the subset of human marker pairs that have a one-to-one match to an ortholog pair in each of the individual three annelid species, as previously inferred [66].

### Amplification of H2A.X variants in *O. fusiformis*

To validate the RNA-seq data and further confirm the expression of the *h2ax1* (*h2ax-y*) and *h2ax2* (*h2ax-f*) genes, we used four different combinations of two forward (Fw) and two reverse (Rv) primers (Additional file 2: Table S16) per gene to selectively amplify the transcript from a cDNA pool obtained from a mixture of developmental stages from *O. fusiformis* ranging from the zygote to the juvenile adult. For each gene, 1 µl cDNA was mixed with 1 µl of 10 µM Fw primer (1 or 2), 1 µl of 10 µM Rv primer (1 or 2), 0.5 µl of 10 mM dNTPs mix, 2.5 µl of 10× ThermoPol reaction buffer, and 0.15 µl of 5000 U·ml^−1^ Taq DNA polymerase, and molecular biology-grade water for a final reaction volume of 25 µl. Samples were amplified by PCR in a thermocycler with a heated lid under the following conditions: initial denaturation at 95 °C for 2 min; 30 cycles of 95 °C for 15 s, 57 °C for 15 s, and 68 °C for 1 min; final extension at 68 °C for 5 min and hold at 12 °C. The length of resulting amplicons (Additional file 2: Table S17) was analyzed via agarose electrophoresis.

### Evolutionary analysis of histone H2A.X variants

An MSA with representative H2A.X sequences across Metazoa (Additional file 2: Table S18) was obtained using MAFFT v.7. [158] as described above. To mine histone H2A.X variants across Eukarya, we performed a protein PHI-BLAST [176] against the non-redundant protein sequences (nr) public database using the H2A.X-Y (search 1) and the H2A.X-F (search 2) orthologs from *O. fusiformis*, using SQ[DE]F and SQ[DE]Y as PHI patterns, for search 1 and search 2, respectively, and a maximum of 1000 target sequences. This ensured the presence of the SQ[DE][FY] carboxyterminal (C-terminal) motif of H2A.X and simplified downstream analysis. Non-eukaryotic sequences were discarded (four for search 1, one for search 2) (Additional file 2: Table S19, S20). Taxonomical information for sequences was obtained using the ETE 3 library [177], and the eukaryotic supergroup was assigned manually based on the assigned phylum and according to current eukaryotic phylogeny [105] (Additional file 2: Table S21–24). Sequences were aligned using MAFFT v.7. as described above. ML phylogenetic inference was performed in IQ-TREE v.2.0.3110 [167] with automatic model selection in ModelFinder Plus. The optimal model was JTTDCMut + R10 [178, 179]. Branch support values were extracted from 1000 ultrafast BS [168]. GraPhlAn v.1.1.3 [180] was used to generate circular representations of the trees. To create sequence logos for the C-terminal motif, H2A.X variants scored against pre-built Hidden Markov Models (HMM) were retrieved from the NCBI Histone Variants Database 2.0 (HistoneDB 2.0) [104] for the Chordata, Streptophyta, Mollusca, Platyhelminthes, and Brachiopoda clades (these last three belonging to the Spiralia clade), aligned using MAFFT v.7. as described above. Alignments were trimmed to the last 12 positions of the alignment, corresponding to the positions 131 to 142 of the histone standardized nomenclature for H2A.X, using Jalview v.2.11.2.6 [181]. Trimmed alignments were turned into information content sequence logos using WebLogo v.2.8.2. [182]. Alignments and the corresponding alignment files in FASTA and Clustal formats are available in the GitHub repository and Zenodo (see Data availability section).

### Histone modifier and reader genes mining and orthology assignment

To mine histone modifier orthologs, we split the search into five different analyses corresponding to the five families of interest: histone deacetylases (HDAC), histone acetyltransferases (HAT), type B HAT, histone demethylases (HDM), arginine-specific histone methyltransferases (PRMT), and lysine-specific histone methyltransferases (KMT) (Additional file 2: Table S25). Gene models corresponding to seven model organisms, namely *H. sapiens*, *M. musculus*, *D. rerio*, *X. tropicalis*, *C. elegans*, *D. melanogaster*, and *Saccharomyces cerevisiae*, were downloaded from NCBI and used to create BLAST local nucleotide databases [160], alongside with the ones corresponding to the three annelid taxa of interest (Additional file 2: Table S26). Histone modifier protein sequences from these seven model organisms (Additional file 2: Table S27) were used as queries to find annelid orthologs following the mutual best BLAST hit approach (*e* value ≤ 10^−5^) (Additional file 2: Table S28–S32), obtaining 67, 97, 120, 40, and 155 unique annelid ortholog candidates for the HDAC, HAT, HDM, PRMT, and KMT families, respectively. HAT type A (nuclear) and type B (cytoplasmic) sequences were split into two groups. Appropriate outgroup sequences were chosen for each of the six alignments (Additional file 2: Table S33). MAFFT v.7 [158] in the L-INS-I iterative refinement method and default scoring parameters were used to generate all six distinct multiple sequence alignments. For orthology assignment, six phylogenetic analyses were performed on selected candidate sequences, which included the longest isoform for each species-gene combination, given that it contained a properly aligned fragment within the family-specific common domain (see below) and that it was not located in a genomic locus of poor sequencing quality. Sequences were trimmed using Jalview v.2.11.2.6 [181] to the family-specific domain(s), i.e., the histone deacetylase (DAC; HDAC), *N*-acetyltransferase (NAT; HAT type A and HAT type B), Jumonji C (JmjC; HDM), *S*-adenosylmethionine methyltransferase protein arginine methyltransferase-type (SAM MTase PRMT-type; PRMT), and the Su(var)3–9, Enhancer-of-zeste and Trithorax (SET; KMT) domains using the domain boundaries defined by ProSITE domain annotation for human HDAC11 (UniProt: Q96DB2; HDAC), KAT2A (UniProt: Q92830; HAT type A), NAA40 (UniProt: Q86UY6; HAT type B), KDM2 A (UniProt: Q9Y2K7; HDM), PRMT1 (UniProt: Q99873; PRMT), and SUV39H1 (UniProt: O43463; KMT) proteins, respectively. Trimmed alignments were used for ML phylogenetic inference with automatic model selection using ModelFinder Plus in IQ-TREE v.2.0.3110 [167]. The optimal models were LG + R7 (HDAC) [178, 183], Q.insect + R5 (HAT type A) [184], LG + G4 (HAT type B) [166], LG + R6 (HDM), LG + F + R5 (PRMT), and LG + R6 (KMT) [179]. Bayesian inference in MrBayes v.3.2.7a [169] was also performed with an LG replacement matrix. Branch support values were extracted from 1000 ultrafast BS [168], and posterior probabilities were estimated from two runs with four chains of MCMC analyses run for 50,000,000 generations (80,000,000 for KMT) (Additional file 2: Table S34). All trees were composed and edited in FigTree v.1.4.4 (http://tree.bio.ed.ac.uk/software/figtree/). The resulting histone modifier orthology assignment is described in detail for each species in Additional file 2: Table S35 and summarized in Additional file 2: Table S36. Alignments and the corresponding alignment files in FASTA and Nexus formats are available in the GitHub repository (see Data availability section).

### PRMT6 sequence and architecture analysis

Candidate PRMT6 sequences were manually selected from a BLAST search of *O. fusiformis*’ PRMT6 protein sequence against the non-redundant (nr) protein database. All ten selected metazoan sequences and the annelid PRMT6 sequences (Additional file 2: Table S37) were aligned using MAFFT v.7 [158] in the L-INS-I iterative refinement method and default scoring parameters. Alignment was trimmed to the conserved region of the protein between residues R43 and Y359, using Jalview v.2.11.2.6 [181], using the nomenclature of human PRMT6 (UniProt: Q96LA8). Residues contained in the PRMT characteristic motif, the SAM-binding pocket, the arginine-binding pocket, and the SAM-interacting and arginine-interacting residues were derived from previous work [111, 112]. Domains and regions with functional relevance were obtained for 11 sequences using InterProScan [185]. Transcriptomic validation of key genes was performed via direct visualization of.bam files in Seqmonk v.1.48.1. Alignment and the corresponding alignment files in FASTA and Clustal formats are available in the GitHub repository (see Data availability section).

### Protein structure prediction

3D structural models of *O. fusiformis* H2A.X-F and H2A.X-Y proteins, as well as of *O. fusiformis* PRMT6, *C. teleta* PRMT6, and *D. gyrociliatus* PRMT6-A, PRMT6-B, PRMT6-C, and PRMT6-D, were created using the AlphaFold Server (https://alphafoldserver.com/about) implementation of AlphaFold 3 [186]. Resulting predictions were rendered in PyMol v.3.0.3 (https://pymol.org/), where the phosphate groups were added to Ser139 and Tyr142 of H2A.X-F and H2A.X-Y, where relevant. Protein structure predictions are available in the GitHub repository (see Data availability section).

### Gene clustering and comparative gene expression analyses

Complete transcriptomes were clustered according to their normalized DESeq2 expression dynamics using soft *k-*means clustering (or soft clustering) in the mfuzz v.2.52 package [187] (Additional file 2: Table S38–S40). Transcripts that were not expressed at any developmental stage were discarded (154 out of 31,979 for *O. fusiformis*, 1242 out of 41,238 for *C. teleta*, and 200 out of 17,387 for *D. gyrociliatus*). The Calinski-Harabasz index [188] was computed to determine an optimal number of 15 (*O. fusiformis* and *C. teleta*) and eight temporally co-regulated gene clusters (*D. gyrociliatus*) using the NbClust v.3.0.1 package [189]. For interspecies comparisons of histone modifiers expression dynamics (Additional file 2: Table S41–S43), clusters were classed as early/cleavage (*O. fusiformis*: 1–5; *C. teleta*: 1–5; *D. gyrociliatus*: 1–2) or late/post-cleavage (*O. fusiformis*: 7–15; *C. teleta*: 7–15; *D. gyrociliatus*: 4–6). Post-cleavage clusters in *O. fusiformis* and *C. teleta* were further subclassified as pre-larval (*O. fusiformis*: 7–9; *C. teleta*: 7–8) and post-larval (*O. fusiformis*: 11–15; *C. teleta*: 11–15). This way, four different quadrants were rendered for each species pairwise comparisons: early_species A_–early_species B_, early_species A_–late_species B_, late_species A_–early_species B_, and late_species A_–late_species B_. Clusters with peak expression in the female adult of *D. gyrociliatus* (7 and 8) were discarded for these purposes. To construct the gene sets of genes under heterochronic shifts, we only considered the histone modifiers with a single-copy ortholog in both *O. fusiformis* and *C. teleta* (for comparisons between *O. fusiformis* and *C. teleta*, Additional file 2: Table S44) or in all three species (for comparisons between *D. gyrociliatus* and *O. fusiformis* or *C. teleta*, Additional file 2: Table S45).

### Acid extraction of histones

Histones were acid-extracted following an adaptation of a previous protocol [190]. 3× *O. fusiformis* adults and 15× *C. teleta* adults were food-deprived for 24 h in artificial seawater (ASW), washed in 1× PBS and spun down for 1.5 min at 3000 × *g*. Animals were homogenized with a pellet pestle motor in 200 µl Tissue Extraction Buffer (TEB) (0.5% Triton X-100, 5 mM sodium butyrate, 2 mM phenylmethylsulphonyl fluoride (PMSF), 0.02% sodium azide, supplemented with 1× cOmplete™ EDTA-free Protease Inhibitor Cocktail Tablets (Roche)). Homogenates were layered over 2.5 ml of a 1.8 M sucrose solution in TEB and centrifuged at 49,000 × *g* and 4 °C for 1 h in a rate-zonal centrifugation. Supernatants were removed, and the pellets were resuspended in 0.5 ml TEB, transferred to a clean tube, and pelleted at 21,000 × *g* and 4 °C for 2 min. Histones were extracted from the nuclear pellets in 1.2 ml 0.5 M HCl for 48 h at 4 °C. Crude extracts were centrifuged at 6500 × *g* and 4 °C for 10 min to remove insoluble debris. 4 × cycles of ultrafiltration in Amicon Ultra-0.5 Centrifugal Filter Units with Ultracel-10 regenerated cellulose membranes (10 kDa nominal molecular weight limit, NWML) (Millipore) using mQ water as exchange buffer were performed following the manufacturer’s recommendations, to remove acid and concentrate histones to 20–25 µl. Aliquots were obtained at various steps to assess protein size and purity via sodium dodecyl sulfate-polyacrylamide gel electrophoresis (SDS-PAGE). For *O. fusiformis*, an additional cleaning step was introduced before the ultrafiltration to remove high molecular weight co-extracted proteins using Amicon Ultra-0.5 Centrifugal Filter Units with Ultracel-30 regenerated cellulose membranes (30 kDa, NWML) (Millipore).

### Histone derivatization and preparation

Histones were prepared for mass spectrometry as previously described [191]. Briefly, histones underwent chemical derivatization by adding 10 μl of 100 mM ammonium bicarbonate pH 8.0 and 4 μl of ammonium hydroxide to 10 μg of histone sample, then adding 10 μl of propionic anhydride in isopropanol and ammonium hydroxide were then used to maintain a pH higher than 8.0. After a 15-min incubation at 37 °C, samples were dried down in a vacuum centrifuge (Concentrator plus, Eppendorf), and the whole process was repeated. Samples were then re-suspended in 40 μl of 100 mM ammonium bicarbonate and digested with trypsin overnight. Digestion was stopped by adding glacial acetic acid and freezing at − 80 °C for 5 min. Samples were vacuum-centrifuged and then underwent two more rounds of proprionylation. Lastly, we used a Hypersep™ Hypercarb™ tip to desalt the samples following the manufacturer’s protocol (Thermo Fisher Scientific) [192].

### Liquid-chromatography tandem-mass spectrometry (LC-MS/MS)

Histone samples were in 0.1% trifluoroacetic acid (TFA) and analyzed on an Ultimate 3000 online nano‐LC system with a PepMap300 C18 trapping column (Thermo Fisher Scientific) coupled to a Q Exactive HF Orbitrap (Thermo Fisher Scientific). Peptides were eluted onto a 50 cm × 75 μm Easy‐spray PepMap C18 analytical column at 35 °C at a flow rate of 300 nl·min^−1^ using a gradient of 3% to 25% over 55 min, and then 25% to 60% until 81 min. Loading solvent was 0.1% TFA and 3% acetonitrile (ACN), solvent A comprised 0.1% formic acid (FA) and 3% ACN, and solvent B was 0.1% FA and 80% ACN. Samples were run in data-independent acquisition mode. Histone posttranslational modifications (hPTM) were identified, and their relative abundance was quantified in EpiProfile 2.0, with manual verification of key hPTMs in Skyline, as previously described [193] (Additional file 2: Table S46, S47).

### Adult gene expression profiling

Publicly available adult RNA-seq datasets for *O. fusiformis* and *C. teleta* [126] (Additional file 2: Table S48) were fetched to explore cross-species differential expression of HMEs, and were processed as described before [126]. DESeq2 v.1.30.1 [175] was used to normalize counts between samples within each species. Those genes with known single-copy orthology between *O. fusiformis* and *C. teleta* as defined in [66], and with a mean TPM value higher than 2, were subset and kept for DESeq2 differential expression analysis. Genes were defined as differentially expressed if they had an absolute log2(fold-change) value higher than 1 and a Benjamini-Hochberg corrected *P*-value lower than 0.05. Differential gene expression results are available in Additional file 2: Table S49.

## Supplementary Information

Additional file 1: Supplementary Figures. Figures S1 to S33.

Additional file 2: 49 Supplementary Tables: Tables S1 to S49.

## Data Availability

Supplementary Figures and Tables are available in the Additional Files 1 and 2, respectively. Accession codes and unique identifiers to previously publicly available datasets we used for this study are listed in Additional file 2: Table S1 (sequence identifiers used in histone genes mining), Additional file 2: Table S2 (sequence identifiers used in histone genes orthology assignment), Additional file 2: Table S8 (ATAC-seq datasets used in chromatin accessibility profiling [194–197]), Additional file 2: Table S9 (RNA-seq datasets used in gene expression profiling [194–200]), Additional file 2: Table S18 (sequence identifiers used in evolutionary analysis of histone H2A.X variants), Additional file 2: Table S26 (genome files used in histone modifier genes mining and orthology assignment), Additional file 2: Table S27 (sequence identifiers used in histone modifier genes mining and orthology assignment), Additional file 2: Table S37 (sequence identifiers used in PRMT6 sequence and architecture analysis) and Additional file 2: Table S48 (RNA-seq datasets used in gene expression profiling in adult stages [201]). The NCBI Histone Variants Database 2.0 (HistoneDB 2.0) can be found at https://www.ncbi.nlm.nih.gov/research/histonedb/. The AlphaFold Server can be accessed at https://alphafoldserver.com/. Updated filtered gene models, transcripts, and protein files, as well as genome annotation reports, alignment files, gene expression files, protein structure predictions, custom code, and any other relevant additional files, are all publicly available in the GitHub repository associated with this study [202] and in Zenodo [203] under an OSI-compliant GNU General Public License v3.0. Mass spectrometry data have been uploaded to PRIDE (accession number: PXD064021) [204].
